# Application of a semivariogram based on a deep neural network to Ordinary Kriging interpolation of elevation data

**DOI:** 10.1371/journal.pone.0266942

**Published:** 2022-04-22

**Authors:** Yang Li, Zhong Baorong, Xu Xiaohong, Liang Zijun

**Affiliations:** 1 School of Geosciences, Yangtze University, Wuhan, Hubei, China; 2 School of Computer Science, Yangtze University, Jingzhou, Hubei, China; 3 Data Company of Xinjiang Oilfield Company of PETROCHINA, Karamay, Xinjiang, China; Military Institute of Science and Technology, BANGLADESH

## Abstract

The Ordinary Kriging method is a common spatial interpolation algorithm in geostatistics. Because the semivariogram required for kriging interpolation greatly influences this process, optimal fitting of the semivariogram is of major significance for improving the theoretical accuracy of spatial interpolation. A deep neural network is a machine learning algorithm that can, in principle, be applied to any function, including a semivariogram. Accordingly, a novel spatial interpolation method based on a deep neural network and Ordinary Kriging was proposed in this research, and elevation data were used as a case study. Compared with the semivariogram fitted by the traditional exponential model, spherical model, and Gaussian model, the kriging variance in the proposed method is smaller, which means that the interpolation results are closer to the theoretical results of Ordinary Kriging interpolation. At the same time, this research can simplify processes for a variety of semivariogram analyses.

## Introduction

Of the many available Kriging methods for spatial interpolation, Ordinary Kriging (OK) [1–3] is considered to be the best linear unbiased estimation method. The fitting effect of the semivariogram [1, 4, 5] of the OK method has a significant impact on the interpolation accuracy [6]. The commonly used semivariogram models include the Gaussian, spherical, and exponential models [7]. In OK interpolation, the optimal selection is generally obtained by comparing many semivariogram models [8–12], and then the optimal model is obtained by manual parameter adjustment. To improve the goodness of fit of the semivariogram model, the least square method [13–15], the maximum likelihood method [16] and the maximum-minimum method [17] can be used to fit the semivariogram function. At present, there are two problems related to semivariogram fitting methods. First, it is necessary to manually analyze and compare the semivariogram to select the appropriate semivariogram model. Second, the least square and maximum likelihood methods for improving the goodness of fit of the semivariogram are limited by the selected semivariogram model. Jo H. and Pyrcz MJ. [18] proposed the use of a convolutional neural network (CNN) to fit the semivariogram. Although this approach realizes automatic fitting and improves the goodness of fit, it relies on a large number of samples and is not suitable for small sample set.

To reduce the cumbersome process of manually selecting the semivariogram model, improve the goodness of fit of the semivariogram model, a deep neural network (DNN) was proposed to simulate the semivariogram for interpolation. Theoretically, the DNN can effectively fit any function [19, 20], including semivariograms. Because multiple learning layers are defined, and multiple neurons are defined in each learning layer, the DNN is obviously more effective than the ANN in fitting the semivariogram. The most important condition that the semivariogram must satisfy is the first law of geography [21–23]. This law shows that the correlation between similar things is stronger than that between dissimilar things, which can be satisfied by the semivariogram simulated by the DNN; that is, the semivariogram simulated by the selected sample points must be an increasing function or an approximately increasing function. By calculating the gradient of the function simulated by the DNN, we can determine the increase and decrease of the variation function at the sample point. Based on the smoothing effect, the traditional semivariogram [24, 25] is unable to fully reflect the real situation, while the semivariogram fitted by the DNN can accurately describe the relationship between things. In this study, a DNN was used to simulate the semivariogram, and the OK interpolation results were compared with the results of the exponential and Gaussian models to verify the effectiveness and rationality of the proposed method.

## Fitting of semivariograms

In this study, Keras of TensorFlow based on Python was used to establish a DNN model for semivariogram fitting. The experimental data were obtained from http://www.gscloud.cn/ (52.635N, 58.748W). Elevation data covering an area of 50 pixels by 50 pixels were selected for testing. Elevation data for 100 points were obtained by uniformly sampling the dataset. A dataset of 2500 pixels was obtained by interpolation and verified by comparison with the original dataset. An isotropic random field was assumed for the dataset used in this study. The r (Eq 1) and h (Eq 1) values of 20 data points were obtained by uniform sampling of 100 data points and used to fit the semivariogram model m (Eq 1). h (Eq 1) represents the distance between any two sample points, and r (Eq 1) represents half of the square of the difference between the values of any two sample points. y (Eq 1) is the gradient, which in this study, refers to the increase or decrease in (h,r). The increase or decrease in the semivariogram is determined by y (Eq 1).

$$\begin{array}{c} y=r^{\prime }=m^{\prime }\left(h\right). \end{array}$$ (1)

**Fig 1:**
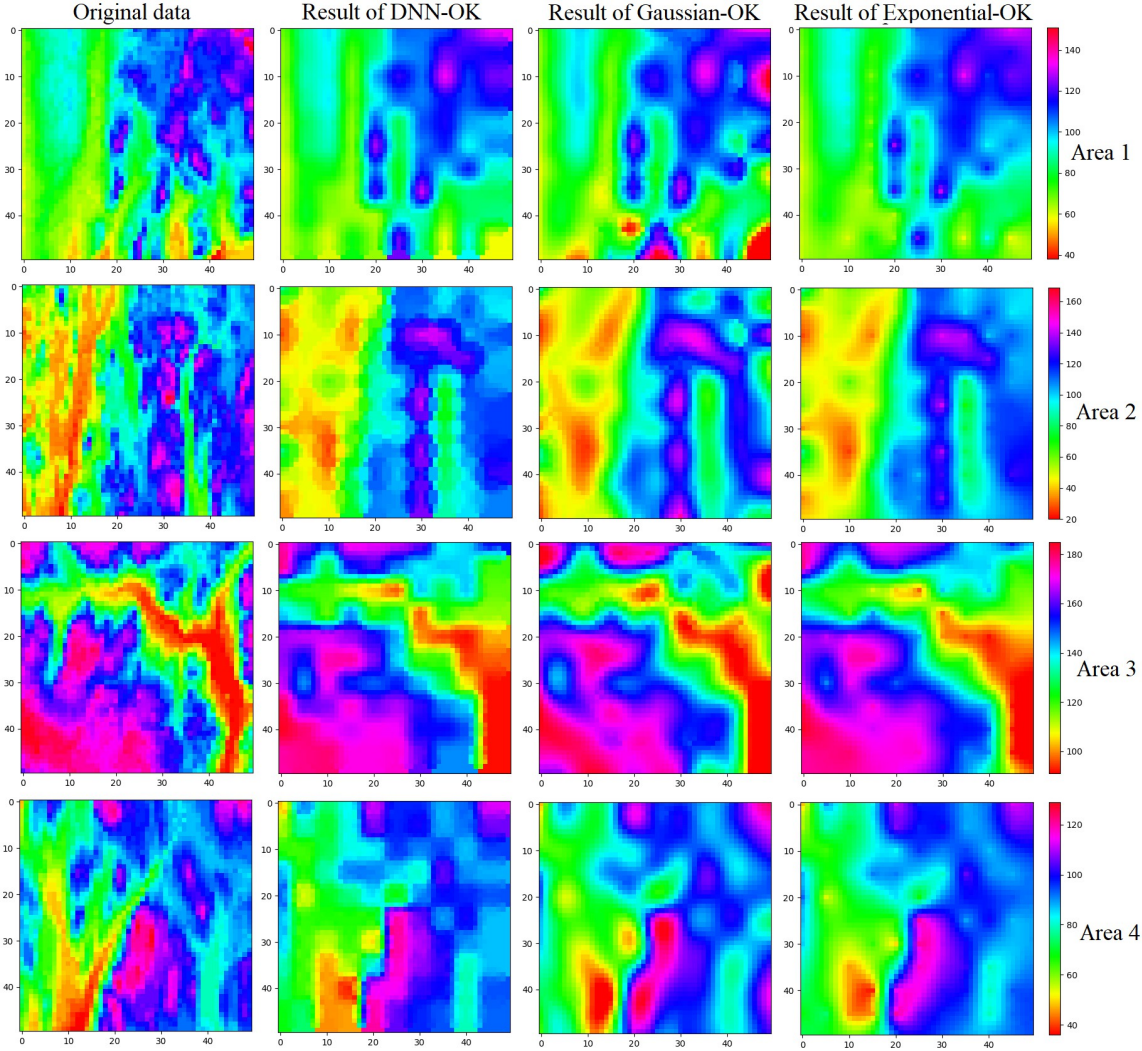
Semivariogram fitting of the DNN model with different training times.

The process of fitting the semivariogram model m (Eq 1) is as follows:

1. Set the DNN training parameters: Set 15 fully connected layers and set the activation function to Relu. The input and output layer activation function is Softplus. The input and output layers have weights of 1. The setting initialization scheme for the other weights is he-normal. Set the input and output layer neurons to 1 (to ensure that the input and output are one-dimensional data). The optimizer is Adam, the learning rate is 0.001, the loss function is the MSE, and the evaluation function is the accuracy and the MSE.
2. The semivariogram must obey the first law of geography, so it is limited to an incremental function. The gradient of a sample point determines the increase or decrease at that point. Set h (input layer) and r (output layer) as the DNN training data. During model training, each training occurs 1 times, and the gradient value y of the model (m in Eq 1) is calculated to determine if y has a negative value. When the gradient value y (Eq 1) appears negative, the model for which the last gradient value y is all positive is selected (to ensure that the semivariogram is the best fit and an increasing function). Fig 1 shows the effect of training 10, 50, 100, and 150 times, including the gradual approach of the curve to the true distribution of the data.
3. The model with k-fold cross-validation [26, 27] distinguishes the training data set and validation data set. The data set of the fitting semivariogram is divided into 10 parts (k = 10), with a total of 10 training iterations. In every training, one part is used as the validation data set, and the other 9 parts are used as the training set to obtain as much effective information as possible in the limited data and optimize the weight of the model.

**Fig 2:**
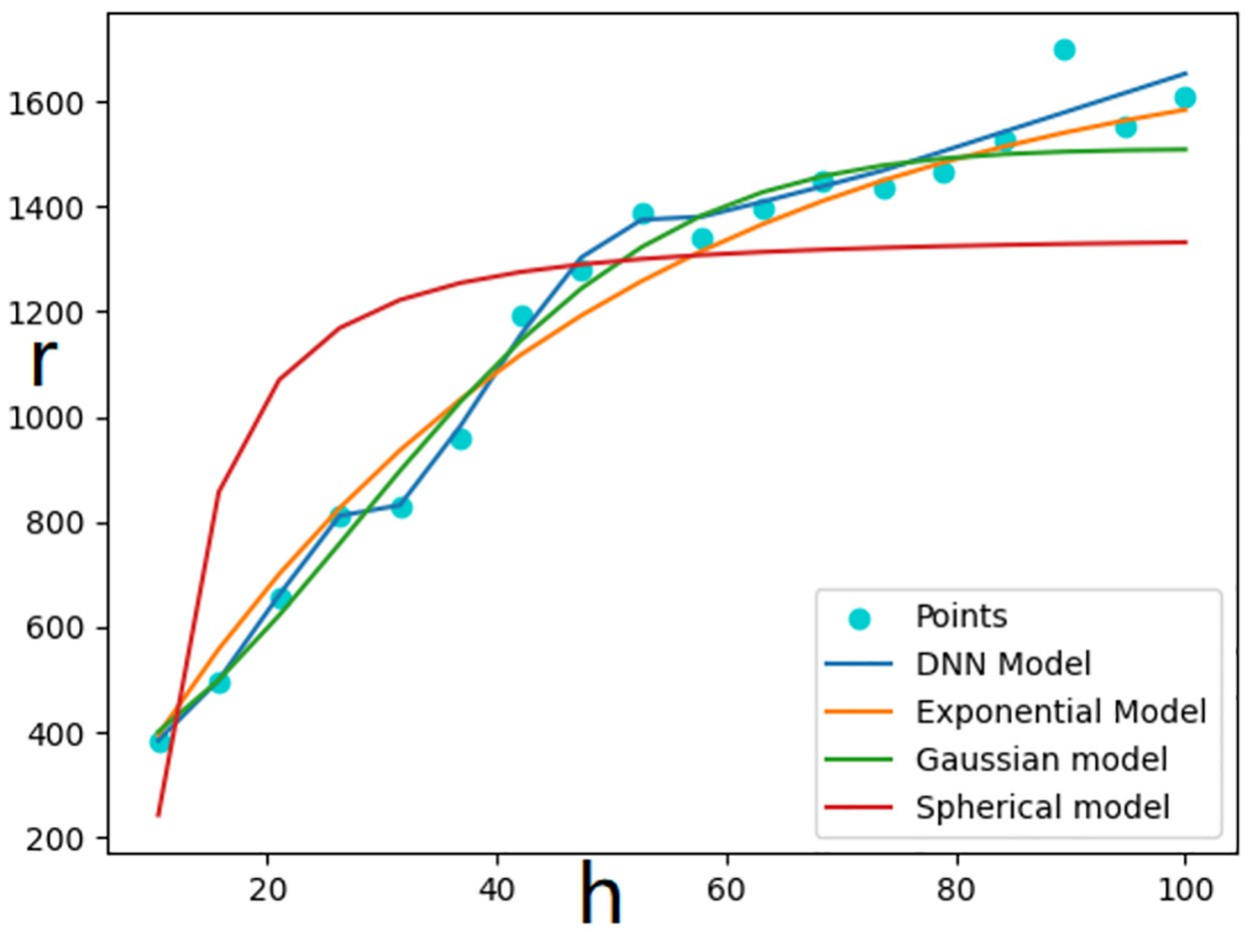
Relationship between training times and fully connected layers.

Compared with ANNs, DNNs have more fully connected layers. The influence of the number of fully connected layers on the training times is shown in Fig 2. Thus, multiple fully connected layers can accelerate the fitting speed of the semivariogram.

**Fig 3:**
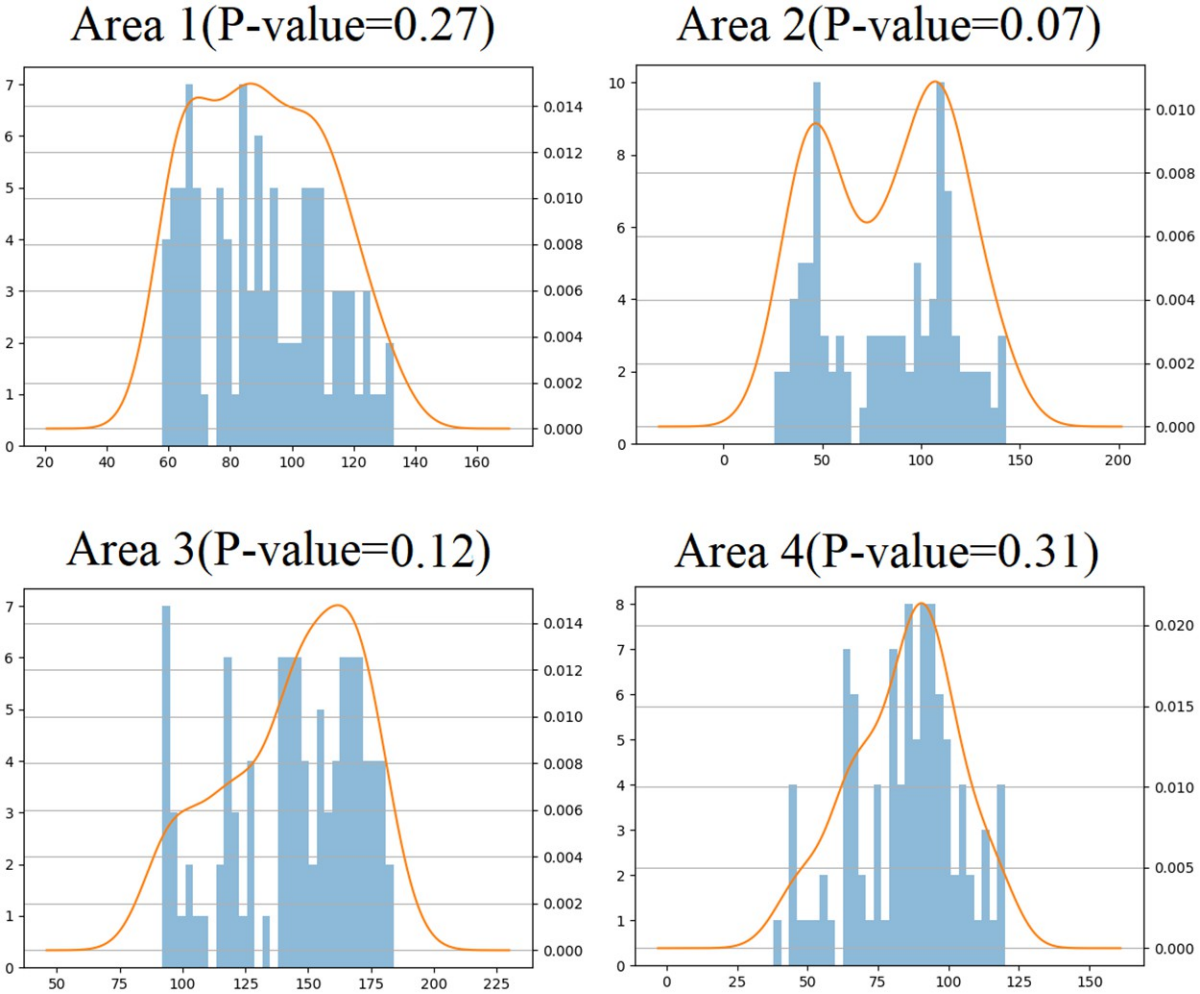
Comparison of fitting results obtained using different semivariogram models.

The rationality of the semivariogram fitted by the DNN was verified by comparison against that of the semivariograms fitted using the spherical, exponential, and Gaussian models. Fig 3 shows that the fitting results of the spherical model were poor, while those of the exponential, Gaussian, and DNN models were extremely similar.

Based on the test of many different DEM data, the following conclusions were obtained for the parameter selection of the initial DNN model based on the proposed method:

1. If the learning rate is too low, the fitting accuracy will be improved, but the training speed will be reduced.
2. As the number of fully connected layers increases, the ease of semivariogram fitting increases, and the number and length of the training times decrease. However, the training time will not be reduced when there are more fully connected layers to a certain extent. The relationship between the number of fully connected layers and the training time will vary according to different data.
3. The activation function is Relu. Relu increases the neuron fitting speed but is prone to neuron death, and the weight cannot be updated. Therefore, the input layer and output layer activation function is set to SoftMax. If all activation functions are Softplus, the effect can be achieved, but the training speed will be very slow. Linear, Sigmoid and Tanh activation functions are not suitable for nonlinear regression according to the algorithm principle.
4. The weight of the input and output layers is initialized to 1 (to prevent gradient disappearance caused by Relu), and the other initialization schemes are he-normal, which can increase the training speed. When the weight is initialized to 0, the training speed is particularly slow. Lecun and Glorot initialization schemes may cause gradient disappearance or gradient explosion, resulting in under-fitting.
5. The optimizer is Adam in the proposed method. Adam is an improvement on RMSprop and Momentum. Adam shows good effects and a short training time. SGD needs to rely on minimal learning rates to achieve good results, and the training time is long.
6. Common loss functions such as the mean square error, mean absolute error, mean squared logarithmic error, and mean absolute percentage error functions produce small differences in this research.
7. To improve the model accuracy, the accuracy and the mean absolute error are used as the evaluation function.
8. Due to the randomness of the deep learning training model, after testing a large number of samples, the training results are slightly different each time. The mean absolute error(MAE) and root mean square error(RMSE) values between the interpolation result and the original figure are less than 0.2.

## OK interpolation

Kriging is one of the main techniques used in geostatistics. A spatial correlation range is determined, and interpolation is performed between data points based on the sampling points within this range. A semivariogram is used to perform unbiased and optimal estimation of attribute values within a limited range and in conjunction with correlation analysis to spatially correlate variables within a specified range. Therefore, kriging produces linear unbiased and optimal estimates for unknown data points based on real data for variables within a specified range and the structural characteristics of a semivariogram. The most significant advantage kriging offers over other interpolation methods is the minimization of the calculation variance.

Cressie [1] provided the derivation of the kriging method. When the sum of the weights (λ) of sample points to unknown points in the kriging method is 1, the method is Ordinary Kriging. The OK interpolation process of a DNN-simulated semivariogram is given as follows:

1. According to the neighborhood search strategy, n known sample points are selected to simulate the semivariogram using the DNN. First, the r (Eq 2) and h (Eq 3) values of any two points *z*_*i*_ and *z*_*j*_ are calculated, where h represents the distance between any two sample points, and r represents half of the square of the difference between the values of any two sample points. x (Eq 3) and y (Eq 3) represent the coordinates of the sample points. Then, h (input of DNN model) and r (output of DNN model) are introduced into the DNN model for training. Specific parameters are set according to the previous chapter until the model reaches the optimal fit.
  $$\begin{array}{c} r\left(z_{i},z_{j}\right)=1/2{\left(z_{i}-z_{j}\right)}^{2}. \end{array}$$ (2)
  $$\begin{array}{c} h=\sqrt{{\left(x_{i}-x_{j}\right)}^{2}+{\left(y_{i}-y_{j}\right)}^{2}}. \end{array}$$ (3)
2. The distance between an unknown point and the known sample point is substituted into the DNN model to obtain the value of the semivariogram. m (Eq 4) represents the DNN model, h (Eq 4) represents the distance between unknown points and n known sample points, and r (Eq 4) represents the correlation between unknown points and known sample points (semivariogram value).
  $$\begin{array}{c} R=m\left(H\right),H=[\begin{array}{c} h_{10}\\ h_{20}\\ \cdots \\ h_{n0} \end{array}],R=[\begin{array}{c} r_{10}\\ r_{20}\\ \cdots \\ r_{n0} \end{array}]. \end{array}$$ (4)
3. Calculate the weight λ(Eq 5) of known sample points to unknown sample points using OK interpolation. K (Eq 5) is the semivariogram value of any two sample points calculated according to Eq 2, and D (Eq 5) is the semivariogram value of the calculated unknown point and the known sample point (obtained according to Eq 4).
  $$\begin{array}{c} \lambda =K^{-1}D. \end{array}$$ (5)
  $$\begin{array}{c} \lambda =[\begin{array}{c} \lambda_{1}\\ \lambda_{2}\\ \cdots \\ \lambda_{n}\\ 0 \end{array}],D=[\begin{array}{c} r_{10}\\ r_{20}\\ \cdots \\ r_{n0}\\ 1 \end{array}],K=[\begin{array}{cccc} r_{11} & \cdots & r_{1n} & 1\\ r_{21} & \cdots & r_{2n} & 1\\ \cdots & \cdots & \cdots & 1\\ r_{n1} & \cdots & r_{nn} & 1\\ 1 & \cdots & 1 & 0 \end{array}]. \end{array}$$ (6)
4. Substitute λ (Eq 5) from the previous step into Eq 7 to calculate the value of the unknown point. *Z*_0_ (Eq 7) represents the value of the unknown point, and *Z*_*i*_ (Eq 7) represents the value of n known sample points.
  $$\begin{array}{c} Z_{0}=\sum_{i=0}^{n}\lambda_{i}Z_{i}. \end{array}$$ (7)
5. The kriging variance, which is calculated according to Eq 8, indicates the deviation between the interpolation results and the theoretical results. *Z*_0_ (Eq 8) represents the calculated real value, ${\hat{Z}}_{0}$ (Eq 8) represents the calculated theoretical value, and $var({\hat{Z}}_{0}-Z_{0})$ (Eq 8) represents the degree of deviation between the calculated and theoretical values.
  $$\begin{array}{c} Var\left({\hat{Z}}_{0}-Z_{0}\right)=\left[\lambda_{1},\lambda_{2},\cdots ,\lambda_{n}\right]\;[\begin{array}{c} r_{10}\\ r_{20}\\ \cdots \\ r_{n0} \end{array}]. \end{array}$$ (8)
6. Repeat steps 1-5 to obtain other unknown points.

## Comparative analysis of the results

**Fig 4:**
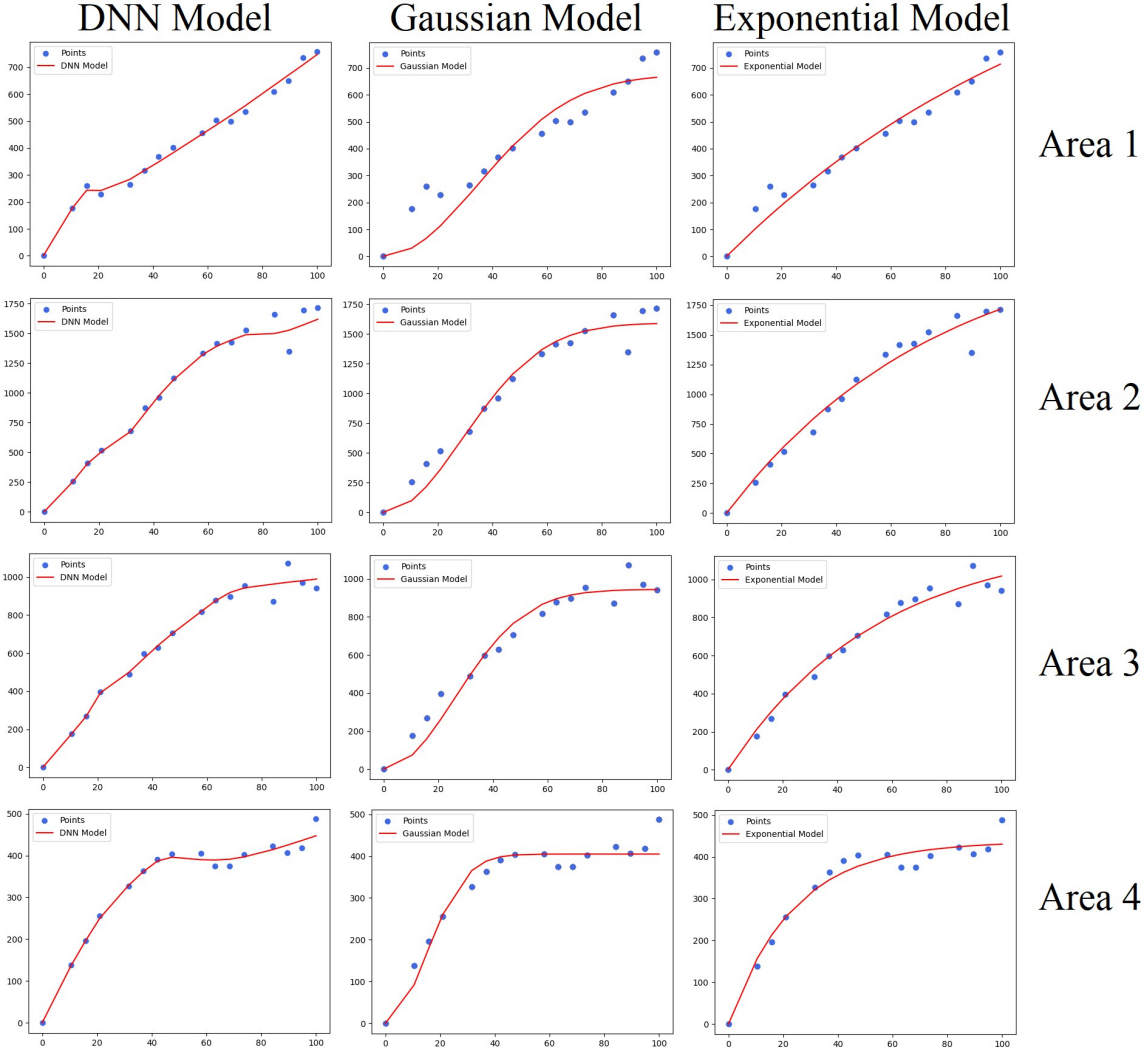
Validation of sample normal distribution in the four areas.

In this study, to verify the rationality of the semivariogram fitted by the DNN, the interpolation results of the fitted semivariogram were compared with those of the Gaussian and exponential models. Before OK interpolation, the normal distribution verification must be carried out for the uniformly selected samples in the four regions. The P-value is obtained through the Kolmogorov Smirnov test. When the P-value is greater than 0.05, the sample set is considered to belong to a normal distribution and is suitable for OK interpolation (Fig 4).

**Fig 5:**
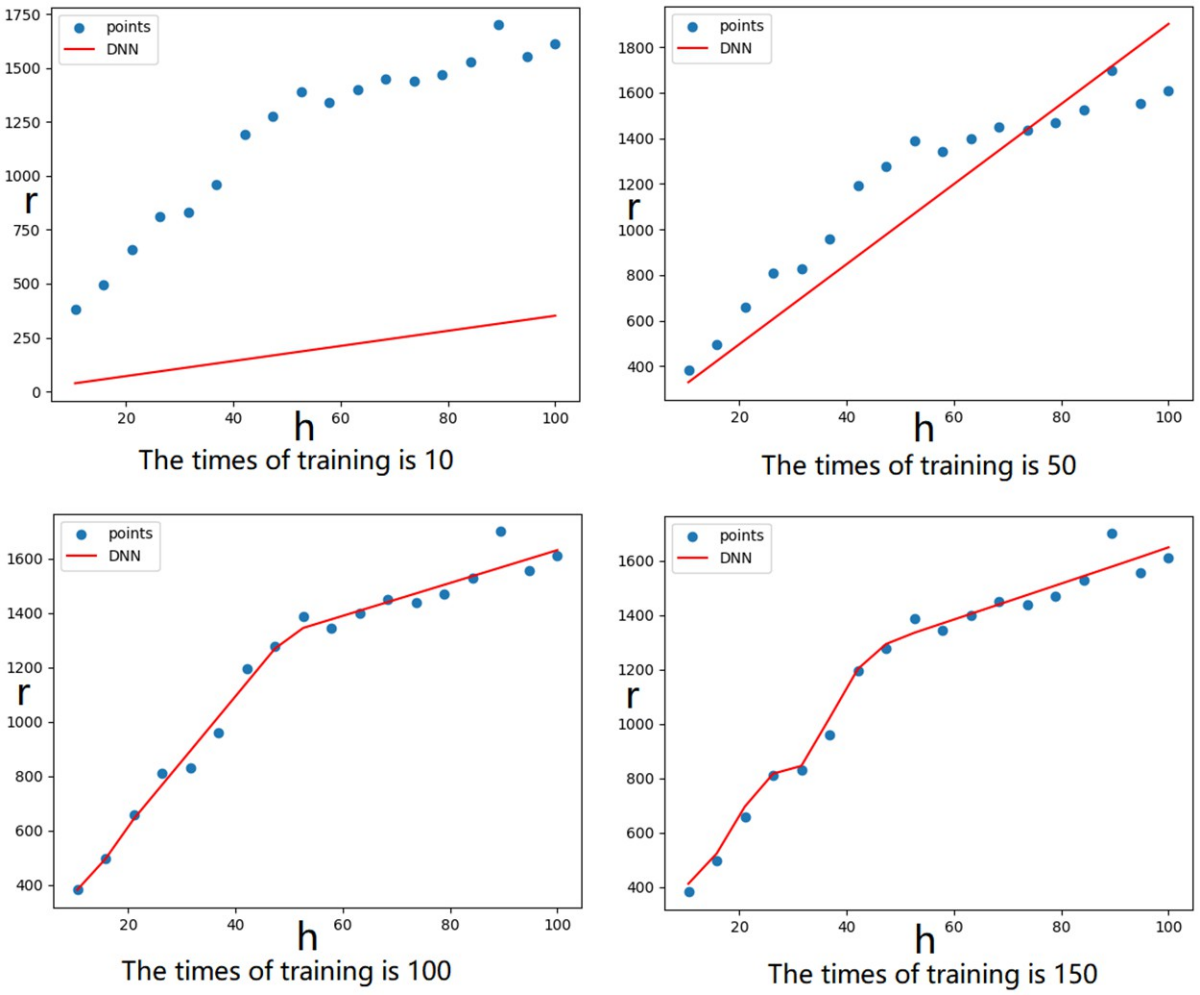
The results of different semivariogram functions in the four areas.

In the data set needed to simulate the different semivariogram models, the 20% of data set are evenly selected as the test set to test the fitting effect of the different semivariogram models, and the remaining 80% of data set are used to fit the different semivariogram models. When the Gaussian model and exponential model are used to fit the semivariogram, the least squares method is used to fit the 80% of data set. When DNN is used to fit the semivariogram, 80% of data set adopt k-fold cross-validation (k = 10) to adjust the weight of the DNN model. Fig 5 shows the fitting effect of different semivariogram models in four areas.

*R*^2^ represents the regression coefficient, which is calculated using the r2_score of sklearn Library in Python. *R*^2^ reflects the proportion of all variations of dependent variables that can be explained by independent variables through regression relationships. The larger *R*^2^ is, the better the fitting effect of the semivariogram is. Table 1 shows the *R*^2^ obtained by fitting the semivariogram with different methods with the 80% of data set in the four areas. Table 2 shows the *R*^2^ obtained by bringing the 20% of data set as test set into different semivariogram models in the four areas. Tables 1 and 2 show that the semivariogram of the DNN simulation has achieved a good fitting effect.

**Table 1:** *R*^2^ obtained by fitting the semivariogram with different methods with the 80% of data set in the four areas.

*R*^2^(80% of data set)	Area 1	Area 2	Area 3	Area 4
DNN model	0.9922	0.9828	0.9845	0.9863
Gaussian model	0.8822	0.9635	0.9588	0.9259
Exponential model	0.9624	0.9697	0.9763	0.9602

**Table 2:** *R*^2^ obtained by bringing the 20% of data set as test set into different semivariograms in the four areas.

*R*^2^(test set)	Area 1	Area 2	Area 3	Area 4
DNN model	0.9842	0.9515	0.9445	0.8594
Gaussian model	0.8223	0.9103	0.8042	0.4519
Exponential model	0.9376	0.9457	0.9382	0.8455

The kriging variance can be used as a standard to evaluate the interpolation results by indicating the degree of deviation from the theoretical results. Therefore, when OK interpolation is performed in unknown areas, the small kriging square difference is helpful to evaluate the interpolation effect. Table 3 shows the mean theoretical kriging variance of the four regions. DNN-OK refers to OK interpolation using the DNN to simulate the semivariogram, Gaussian-OK refers to OK interpolation using the Gaussian model to simulate the semivariogram, and Exponential-OK refers to OK interpolation using the exponential model to simulate the semivariogram. Table 3 shows that the semivariogram simulated by the DNN produces a small kriging variance.

**Table 3:** Comparison of the mean of the kriging variance in the four areas.

The kriging variance	Area 1	Area 2	Area 3	Area 4
DNN-0K	52.52	75.22	131.69	73.53
Gaussian-0K	90.87	84.95	140.46	85.60
Exponential-0K	92.42	90.37	144.56	87.01

**Fig 6:**
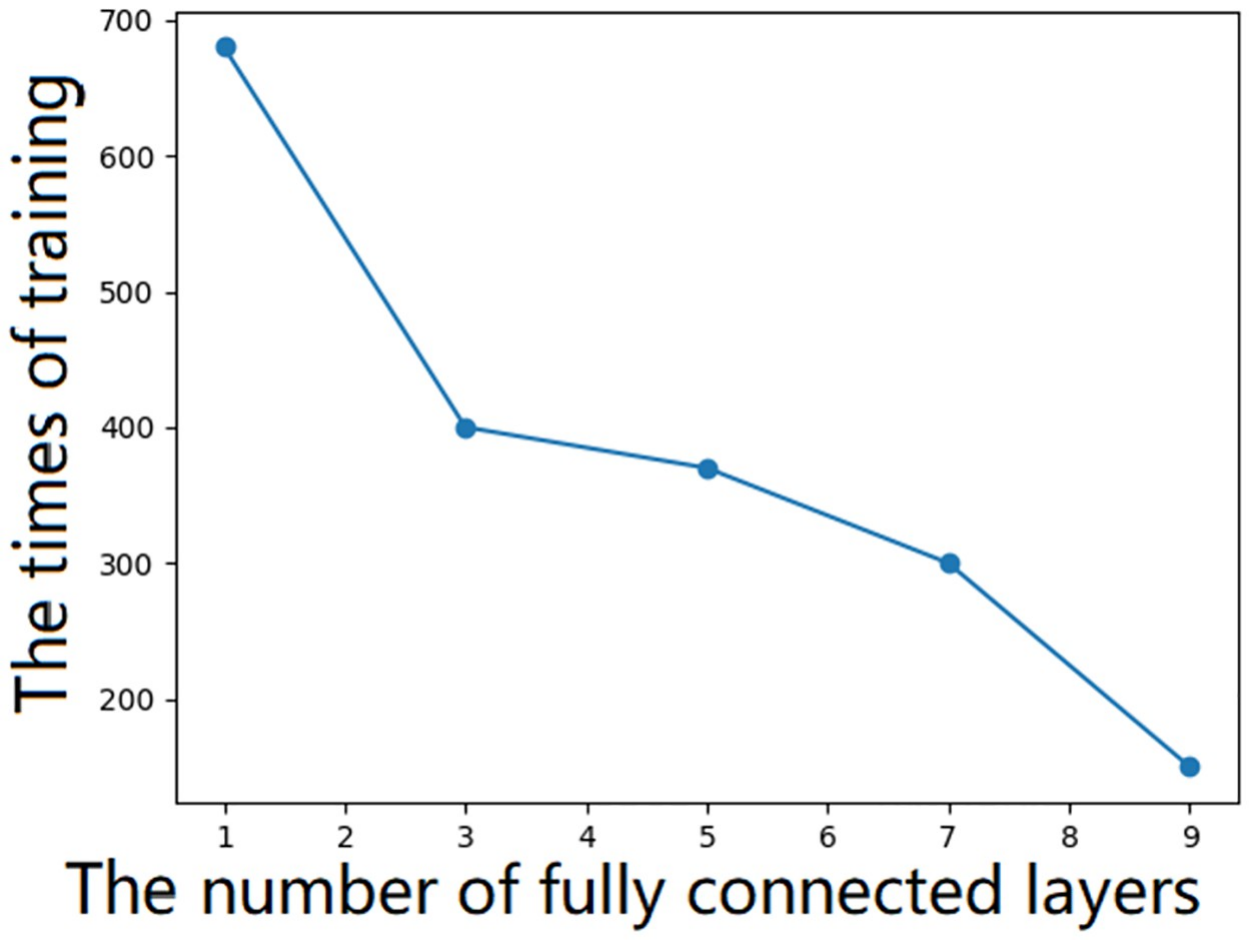
Comparison of fitting results obtained using the different models in the four areas.

Tables 4 and 5, and Fig 6 show the OK interpolation results of the different semivariograms in the four regions. The mean absolute error (MAE, Eq 9) and root mean square error (RMSE, Eq 10) values are used for error analysis. In Eqs 9 and 10, ${\hat{Z}}_{i}$ represents the interpolation result, and *Z*_*i*_ represents the actual value in the original data. Tables 4 and 5 show the MAE and RMSE values between the Gaussian-OK, Exponential-OK, DNN-OK interpolation results and the original data in the four areas. The DNN-simulated semivariogram clearly improves the accuracy of OK interpolation.

$$\begin{array}{c} E_{MAE}=|\frac{1}{n}\sum_{i=1}^{n}\left({\hat{Z}}_{i}-Z_{i}\right)| \end{array}$$ (9)

$$\begin{array}{c} E_{RMSE}=\sqrt{\frac{1}{n}\sum_{i=1}^{n}{\left({\hat{Z}}_{i}-Z_{i}\right)}^{2}} \end{array}$$ (10)

**Table 4:** MAE between the interpolation results and the original data in the four areas.

MAE	Area 1	Area 2	Area 3	Area 4
DNN-0K and original data	6.0270	10.6652	7.7237	5.5069
Gaussian-0K and original data	8.1987	12.2126	8.9987	5.7938
Exponential-0K and original data	6.1087	10.8412	7.7573	5.6161

**Table 5:** RMSE between the interpolation results and the original data in the four areas.

RMSE	Area 1	Area 2	Area 3	Area 4
DNN-0K and original data	9.3568	14.8999	12.8717	7.8355
Gaussian-0K and original data	14.1523	17.1283	16.4379	8.0862
Exponential-0K and original data	9.1970	14.9906	12.9399	7.8406

The following conclusions can be drawn from Tables 1–5, Figs 5 and 6:

1. The DNN-simulated semivariogram can replace the semivariograms based on the exponential and Gaussian models.
2. The interpolation results of the DNN-simulated semivariogram improve the accuracy of OK interpolation and reduce the theoretical kriging variance.

The causes of errors are as follows:

1. Different semivariogram models produce different goodness of fit values, which affects the interpolation accuracy.
2. OK interpolation is carried out on the premise of a standard normal distribution, but the samples are only approximately normally distributed. Generally, if the P-value is greater than 0.05, a distribution is considered to be normal, which is one of the reasons for the interpolation accuracy.

## Conclusion

In principle, a DNN can be used to fit all functions; thus, a novel method for fitting semivariograms using a DNN was proposed in this study. The following conclusions were drawn from the study results.

1. A semivariogram is mainly used to reflect spatial correlation. In traditional OK interpolation, various semivariograms (Gaussian model, exponential model, spherical model, etc.) must be analyzed, and the best fitting semivariogram must be selected to reflect spatial correlation. However, these semivariograms have specific models and cannot summarize all geographical distributions. A DNN can theoretically fit all functions. Under the premise of the first law of geography, this capability means that the best fitting semivariogram can be obtained, reflecting the optimal spatial correlation. The advantage of this method is that it can replace most other semivariogram functions and simplify the process of analyzing various semivariogram functions.
2. The kriging variance, which can be used as a criterion to evaluate the interpolation results, represents the degree of deviation from the theoretical results. Therefore, in the case of an unknown spatial distribution, a small kriging variance is helpful to evaluate the kriging interpolation effect. The experimental results show that the best semivariogram fitting has the smallest kriging variance, which means that the results calculated by this method are closer to the theoretical interpolation results. The proposed method optimizes the OK algorithm.
3. Compared with the traditional semivariogram model, the semivariogram based on DNN fitting requires more computing time, so the GPU performance must be improved to save time. However, this approach can save artificial analysis time compared to traditional artificial selection methods for semivariogram analysis.
